# Steroid-Modified Dermatophytosis: A Clinical and Diagnostic Challenge

**DOI:** 10.7759/cureus.108993

**Published:** 2026-05-16

**Authors:** Vaishnavi Jadhav, Priyanka M Mane, Satish R Patil

**Affiliations:** 1 Department of Microbiology, Krishna Institute of Medical Sciences, Krishna Vishwa Vidyapeeth (Deemed to be University), Karad, Maharashtra, IND

**Keywords:** clinical challenges, dermatophyte, diagnostic challenges, steroid-modified tinea, superficial mycoses, tinea incognito

## Abstract

Dermatophytosis represents one of the most prevalent superficial fungal infections affecting the skin, hair, and nails worldwide. In recent years, the widespread and often indiscriminate use of topical corticosteroids has been frequently combined with antifungal and antibacterial agents. This narrative review explores the impact of corticosteroid misuse on dermatophytosis, focusing on changes in pathogenesis, clinical manifestations, diagnostic challenges, management, and treatment. Special emphasis is placed on the emerging epidemic of steroid-modified dermatophytosis in India, driven by over-the-counter drug availability and irrational prescribing practices, leading to chronic, recurrent, and recalcitrant cases. Understanding these effects is essential for microbiologists and clinicians to enhance antifungal stewardship, treatment outcomes, and diagnostic precision.

## Introduction and background

Dermatophytes are keratinophilic fungi that cause superficial infections of keratinized tissues, such as the skin, hair, and nails [1,2]. They are members of the genera *Trichophyton, Microsporum*, and *Epidermophyton*. Together, these infections are referred to as dermatophytosis, or "tinea," and are among the most prevalent fungal infections worldwide [2]. Dermatophytosis represents a major global public health problem. It affects people of all ages and income levels. The burden is especially heavy in tropical and subtropical areas, where warm, humid weather makes it easier for fungi to grow and spread [3]. In the past decade, dermatophytosis has undergone a significant epidemiological shift, particularly in tropical nations like India [3,4]. This shift means that more cases are chronic, recurrent, and hard to treat, which is a big problem for doctors. There are many reasons this happened, including poor hygiene, overcrowding, and limited access to appropriate treatment [1].

The abuse of topical corticosteroids, which are frequently found in creams that combine antifungal and antibacterial ingredients, is primarily responsible for this shift [5,6]. These irrational fixed-dose combinations are often sold over the counter and are often misused without a doctor's supervision, which makes the problem worse [3]. Corticosteroids alter the function of the skin barrier, suppress local immune responses, and conceal traditional clinical symptoms, such as scaling and erythema [6,7]. Consequently, infections become persistent, unusual, and unresponsive to conventional antifungal treatment; this condition is now known as steroid-modified dermatophytosis or tinea incognito [8,9].

In addition, the inappropriate use of corticosteroids results in unusual morphologies, which complicates clinical diagnosis and can delay effective treatment. Understanding the interplay between steroids and dermatophytes is critical for improving management strategies. Additionally, the development of antifungal drug resistance, especially in *Trichophyton *fungi, has made the problem worse, necessitating a change in therapeutic strategies [6].

## Review

Methods

Search Strategy

**Figure 1:**
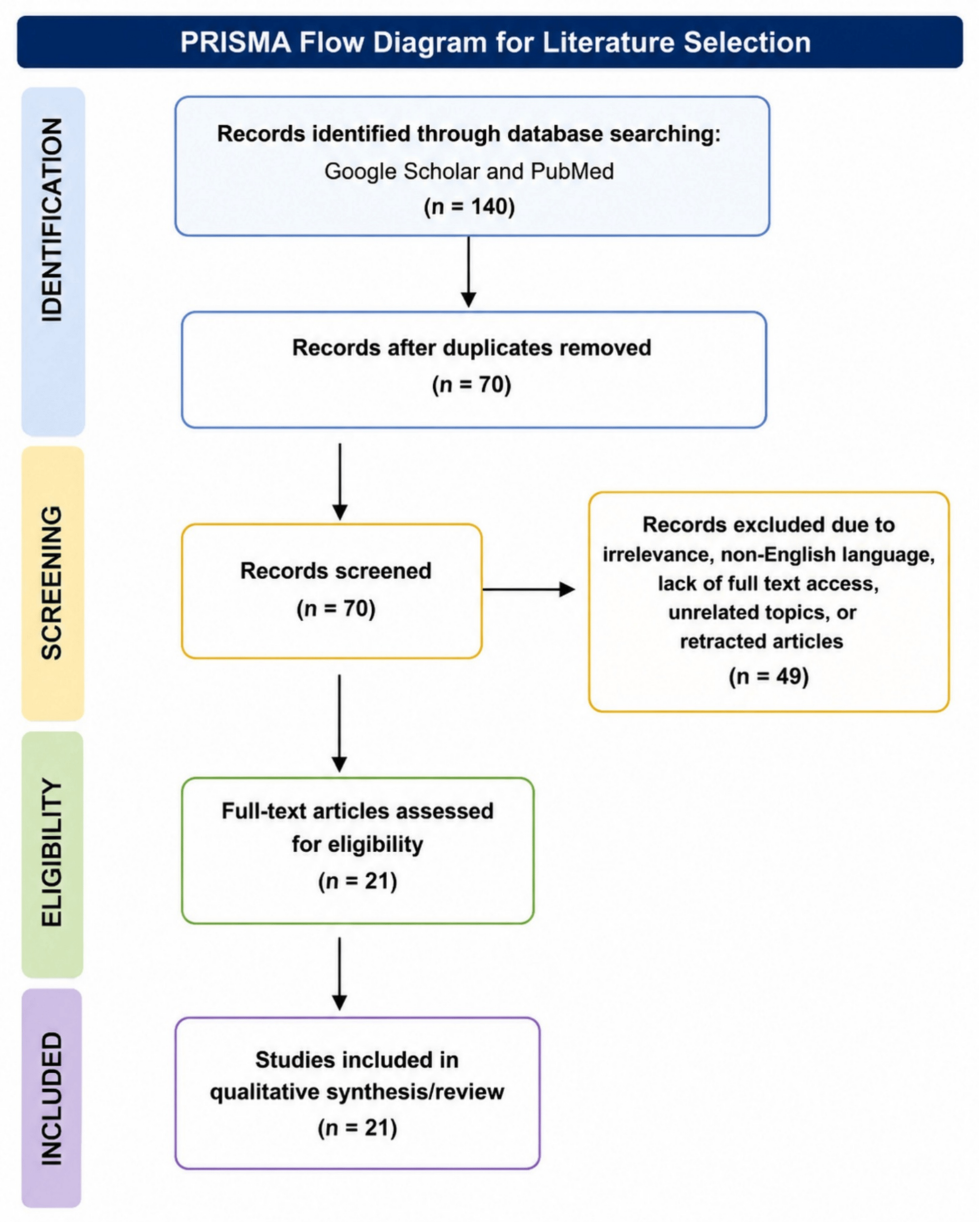
Article selection and screening process

To prepare this narrative review article, a comprehensive literature search was conducted using electronic databases such as Google Scholar and PubMed. The search covered from 2014 to 2026 and utilized relevant Medical Subject Headings (MeSH) terms and keywords, including “steroid-modified tinea,” “tinea incognito,” “dermatophytosis,” “ringworm infection,” “topical steroid misuse,” “diagnosis of tinea,” and “treatment of dermatophytosis.” These terms were used individually and in combination using Boolean operators to refine the search. Research publications that did not meet the inclusion criteria, including studies not written in English or those unrelated to steroid-modified dermatophytosis, diagnosis, treatment, or antimicrobial resistance (AMR), were excluded, along with retracted articles and studies lacking full-text access. The initial database search yielded 140 articles for analysis. After the removal of duplicate records and irrelevant studies, 70 articles remained for further screening. Following detailed evaluation based on the identification, selection, inclusion, and exclusion criteria, 21 articles were ultimately included in the final review (Figure 1). The evidence-based discussion presented in this review was developed through a comprehensive analysis of the selected literature.

Epidemiology

The condition known as tinea incognito is a less-known type of dermatophytosis that presents unusual and sometimes deceptive clinical presentations owing to the misuse of corticosteroids and other immunosuppressing drugs both topically and systemically. Its true prevalence rate cannot be determined due to the fact that it is frequently misdiagnosed for eczema, psoriasis, or other inflammatory skin diseases; nevertheless, there has been an observable increase in cases reported across the globe [7-9]. This rise is especially noticeable in developing nations like India, where steroids in combination with various other drugs are easily available [3].

According to epidemiologic studies, there appears to be an increased frequency of occurrence of tinea incognito among populations residing in tropical and subtropical countries marked by high levels of heat and humidity, creating favorable conditions for fungal growth and transmission [1,3]. Low standards of hygiene, crowding, and personal contact between individuals contribute to the prevalence of the disease, while steroid misuse, which leads to the formation of unusual manifestations, becomes more prevalent under such conditions. All age groups may become targets of tinea incognito; nevertheless, adults are the largest proportion of patients because of higher frequencies of self-treatment using topical preparations [5,7].

Some researchers have emphasized that tinea incognito accounts for a considerable percentage of chronic and recurring cases of dermatophytosis [3]. This type of dermatophytosis is usually linked to infections resulting from the *T. mentagrophytes *complex and *T. rubrum*, which are the common dermatophyte pathogens responsible for the ongoing dermatophytosis epidemic in India and elsewhere [7,9]. In addition to affecting the appearance of the lesions, the inappropriate use of steroids leads to suppressed immune responses and deep fungal invasion. Another aspect of the epidemiology of tinea incognito that merits consideration is the development of resistance against antifungal agents in the dermatophytes. The chronic nature of the infection, improper treatment regimens, and the use of combination drugs such as antifungal and steroid creams lead to treatment failure and recurrences [7-9]. All these factors, coupled with patient and general practitioner ignorance, have contributed to the rise in the incidence of tinea incognito, necessitating its recognition as a significant health concern.

Pathogenesis

Steroid-modified tinea occurs when a dermatophyte infection is exposed to topical and systemic steroids, resulting in immunosuppression and modification of the primary presenting features [7,10]. Pathogenesis involves a complex sequence of events, including invasion, immunomodulation, immune masking, and proliferation. Infection begins with the entry of dermatophytes into the stratum corneum due to the use of keratinases, lipases, and proteases, which break down keratin [1]. This early infection usually occurs after an inflammatory reaction due to the host's immune response, which is usually characterized by hyperproliferation followed by innate and adaptive immunological reactions, including responses mediated by Th1 and Th17 lymphocytes [11].

When using topical steroids, they inhibit this inflammatory reaction vastly. Corticosteroids suppress the production of proinflammatory cytokines, such as IL-1, IL-2, IL-12, and TNF-α; inhibit neutrophil migration; and reduce macrophage activation [6]. This results in the inhibition of cell-mediated immunity, which is regarded as the most potent defense mechanism against dermatophytes, resulting in the sustained growth of fungi in the skin [7,11]. They also alter host immune responses by suppressing Th1- and Th17-mediated pathways that are essential for fungal clearance. In addition to reducing fungal elimination, corticosteroids diminish the inflammatory response responsible for the characteristic clinical features of dermatophytosis, such as erythema, scaling, and an active border. This results in an altered host-pathogen interaction, wherein fungal elements may persist or extend with reduced visible inflammation, leading to atypical clinical morphology rather than simply increased fungal proliferation. With continued corticosteroid use, progressive modulation of local immune responses leads to a clinically modified infection known as tinea incognito [8,9]. In this condition, the typical ring-shaped border, scaling, and active advancing edge are reduced or absent. Instead, lesions may appear eczematous, psoriasiform, or rosacea-like, often with ill-defined margins, making the diagnosis challenging and potentially misleading [9].

As fungal colonies grow further within an immunosuppressed microenvironment, exaggerated fungal growth and barrier breakdown are observed. The intensification of keratin breakdown, fungal density, deepening of follicular invasion, and chronicity are additive findings. In severe cases, deep invasion may cause the formation of Majocchi’s granuloma [1]. The extensive use of steroid cream combinations for fungal and bacterial infections, especially in India, further contributes to chronic steroid-modified tinea infections and the development of dermatophytes with reduced sensitivity, such as *T.* *indotineae* (previously classified within the *T. mentagrophytes* ITS type VIII complex), which is now recognized as a major terbinafine-resistant dermatophyte associated with the Indian dermatophytosis epidemic [12,13].

Cutaneous lesions caused by dermatophytic infections are also significantly affected by the abuse of topical corticosteroid creams, leading to various unconventional manifestations summarized as "steroid-modified dermatophytosis or tinea incognito" [8]. The typical presentation of dermatophytic infections, which includes well-defined round lesions with prominent erythematous edges and a relatively clear center, may not always occur or may be irregularly modified and may consist of lesions with subtle scales and reduced inflammation due to the strong anti-inflammatory properties of corticosteroids [14].

Clinical manifestations

A common clinical manifestation is the absence of typical morphology [8,9]. The border may be irregular or diffuse, and there may be a lack of central clearing features that may be seen in lesions that tend to involve multiple body areas concurrently and spread in a centripetal pattern [15]. Topical steroid treatment may also be accompanied by cutaneous abnormalities, such as striae, telangiectasias, and hypopigmentation, with fungal infection, which may be more difficult to detect because of these conditions [6,16].

Patients with dermatophytosis modified by steroids often experience marked itching and burning, especially during periods of steroid withdrawal [7]. Rebound inflammation can initiate a cycle of frequent steroid use. Secondary infections may arise from disruption of the epidermal barrier, causing lesions such as oozing, crusting, or pustular lesions. Follicular lesions can occur, manifesting as folliculitis or acneiform eruptions, particularly tinea corporis or cruris [17]. Various dermatophytosis patterns have been reported in patients exposed to steroids. Tinea incognito occurs as eczematoid, psoriasiform, rosacea, or lupus dermatitis, commonly resulting in misdiagnosis [8,9,18]. Tinea pseudoimbricata features many concentric rings with imbricated tinea, which occurs due to the cyclic reduction in the growth of the pathogenic organism because of intermittent steroid intake [17,19]. Large areas of the body or disseminated dermatophytosis, often occurring at multiple sites, have been found in cases of widespread steroid abuse [3].

Additionally, the abuse of potent topical corticosteroids in children and infants poses another risk. Increased cutaneous absorption in infants, owing to the thin skin, may cause systemic side effects, such as iatrogenic Cushing's syndrome, growth suppression, and hypothalamic-pituitary-adrenal axis suppression [6]. Genital and facial lesions, primarily in children and females, are increasingly being observed, often due to the abuse of combined creams for pruritic dermatoses.

The altered clinical presentation of steroid-modified dermatophytosis often masquerades as other inflammatory dermatoses, including eczema, psoriasis, seborrheic dermatitis, or contact dermatitis [7,9]. There is thus a great deal of overlap and diagnostic confusion, with delays in appropriate antifungal therapy. Therefore, patients frequently arrive late with chronic, recurrent, and resistant disease, highlighting the importance of correlating clinical findings with laboratory confirmation. As shown in Table 1, steroid-modified dermatophytosis exhibits altered clinical features compared to classical dermatophytosis.

**Table 1:** Comparison of clinical features between classical dermatophytosis and steroid-modified dermatophytosis

Features	Classical dermatophytosis	Steroid-modified dermatophytosis
Lesion margin	Well-defined	Ill-defined
Scaling	Prominent	Minimal
Erythema	Present	Reduced
Central clearing	Present	Absent
Symptoms	Mild itching	Severe itching/burning
Distribution	Localized	Widespread/multifocal

Laboratory diagnosis

An accurate diagnosis of dermatophytosis is important for appropriate management, epidemiological control, and prevention of the development of antifungal resistance [11]. However, the diagnosis of dermatophyte infections has become increasingly challenging in the modern context of rampant corticosteroid abuse, especially in cases of steroid-modified dermatophytosis [7]. Corticosteroids may mask inflammation and alter lesion morphology, making clinical recognition and sampling more difficult. Tinea incognito is classically described as a dermatophyte infection with atypical features caused by topical/systemic steroids or other immunosuppressive treatments [7].

An accurate diagnosis starts with appropriate specimen collection, which is often the most important and difficult step in the diagnostic process. Skin scrapings, hair stubs, or nail clippings should be obtained from the active margins of lesions, where viable fungal elements are most concentrated. Corticosteroid application suppresses inflammation and scaling, resulting in poorly demarcated lesion borders with meager surface scaling. Thus, fungal elements tend to remain deeper in the stratum corneum, and superficial scraping is insufficient. Previous or concomitant therapy with antifungal agents further reduces fungal viability, leading to an increased incidence of false-negative results. Thus, an improved diagnostic yield often requires cessation of topical agents for several days before sampling and repeat specimen collection, especially in chronic or recalcitrant diseases [7].

To date, direct microscopy with a potassium hydroxide (KOH) mount is still considered the gold standard for initial diagnosis owing to its simplicity, rapid turnaround time, and ease of performance. A 10%-20% KOH mount lyses keratin, allowing easy detection of hyaline, septate, or branching hyphae, which are typical for dermatophytes. Unfortunately, conclusive evidence for dermatophytes is rarely available in patients with steroid-modified dermatophytosis, in whom fungal structures may be few, broken up, or morphologically aberrant, even for experienced microscopy examiners. Steroidal immunomodulatory effects will also lead to diminished keratinocyte turnover, causing diminished keratinous debris and paradoxically limiting fungal structures for observation. Various fixative alternative staining methods have been introduced, namely calcofluor, Chicago Sky Blue, or even Parker ink-KOH, to increase sensitivity and aid in more accurate diagnosis​​​. Even so, microscopy has no dermatophyte-specific cells that can reliably identify dermatophytes from non-dermatophyte molds or yeasts [7].

KOH microscopy is a rapid first-line diagnostic test, whereas fungal culture remains the reference method for definitive identification and species confirmation. The samples are usually placed on Sabouraud Dextrose Agar, which contains chloramphenicol and cycloheximide to prevent bacterial overgrowth and saprophytic fungi. Dermatophyte test medium (DTM) is preferred when screening samples because an alkaline reaction occurs upon dermatophyte growth, and the change in color from yellow to red is observed. The samples are then cultured in an incubated environment at 25°C-30°C and viewed for up to four weeks. However, steroid-modified samples ​​​​​​usually show delayed growth or failure to grow due to decreased viability and low inoculum. There is likely bacterial contamination and mold contamination, especially when there is skin damage due to chronic steroid use [7].

However, even when growth is successful, species identification can be difficult based on colony characteristics ​​​​​and microscopy using Lactophenol Cotton Blue staining. A large overlap has been observed among some species like *T. rubrum, T. mentagrophytes*, and the recently discovered *T. indotineae.* Moreover, differences observed in sporulation, pigmentation, and colony characteristics, particularly among steroid-exposed cultures, also make identification via phenotype increasingly difficult [20].

Recent developments in molecular diagnostics have greatly optimized the rapidity and accuracy of dermatophyte identification.​ Polymerase chain reaction (PCR) tests based on the ribosomal ITS region have enabled the detection of dermatophyte DNA in negative fungal cultures and clinical samples. Real-time PCR analysis also enables species identification and estimation of fungal load in infected tissues. ITS- and β-tubulin-gene sequencing tends to be the backbone in differentiating closely related species and identifying new emerging fungal agents, including *T. indotineae*, among others. Although these methods are highly sensitive and specific, they are expensive, lack standardization, and require infrastructure and processing that restrict practical utility to reference centers in major hospitals [20]. Matrix-assisted laser desorption/ionization-time of flight mass spectrometry (MALDI-TOF MS) represents a promising approach for quickly identifying dermatophytes based on proteomic signatures. Although MALDI-TOF MS provides rapid results once growth is established, its reliance on viable organisms, along with the lack of comprehensive dermatophyte libraries, impedes its widespread use [11].

With the emergence of antifungal resistance, particularly among *T. indotineae* strains to terbinafine and azoles, the role of antifungal susceptibility testing (AFST) has become increasingly important. Several broth microdilution techniques, based on the Clinical and Laboratory Standards Institute (CLSI M38-A2) guidelines and the European Committee on Antimicrobial Susceptibility Testing (EUCAST) directives, are used to determine minimum inhibitory concentrations (MICs). However, variations in dermatophyte growth rates, the absence of defined clinical breakpoint values, procedural complexity, and technical challenges have limited the routine application of AFST in clinical laboratories. Nevertheless, AFST remains essential in the management of chronic, persistent, and treatment-unresponsive dermatophytosis, particularly for monitoring population-level resistance [12,21].

Management and treatment

A comprehensive treatment plan for managing tinea incognito involves discontinuation of improper treatments, diagnosis, and application of antifungal therapy [4,7]. The very first step that should be considered in this case is stopping the application of improper treatments such as topical corticosteroid creams. These treatments cause an impairment in clinical manifestations of tinea incognito, hence facilitating its growth [5-7]. Patients should be taught about the dangers of using steroid-containing preparations without a prescription to prevent recurrence of the problem [5].

After discontinuing the use of steroids, the next step would be initiating antifungal treatment according to the degree of infection. Local application of antifungal drugs, which include azoles (clotrimazole, ketoconazole, and luliconazole) and allylamines (terbinafine and naftifine), is suitable for mild to localized infections [2,4]. The mechanism of action of these drugs involves inhibition of ergosterol synthesis, thereby disrupting fungal cell membrane integrity. Nevertheless, in almost all cases of tinea incognito, oral administration of antifungal drugs is essential since they are widespread, long-lasting, and present with varying appearances. Commonly used drugs include terbinafine, itraconazole, and fluconazole, with terbinafine and itraconazole being more preferable because of their fungicidal actions and high efficiency against dermatophytes [4,11].

The duration of therapy is usually longer than that of common dermatophytosis, and the treatment must be administered until full resolution is achieved clinically and, if possible, mycologically [4,7]. Supportive therapy includes maintaining hygiene, keeping the affected sites dry, wearing non-occlusive garments, evaluating close contacts, and treating clinically or mycologically confirmed infections when appropriate to avoid reinfection [5,6]. When there is a secondary bacterial infection or inflammation following the withdrawal of corticosteroids, short-term administration of anti-inflammatory drugs or antihistamines may be required [4].

The emergence of antifungal resistance, especially with *T. mentagrophytes* complex, has been a serious problem when it comes to the treatment of tinea incognito [11-13]. Treatment failures and cases of relapses have become common occurrences that have led to the use of high doses, extended treatment durations, or combinations of antifungal drugs in cases of resistance. Thus, AFST and prudent use of antifungal drugs are highly recommended. However, early diagnosis, proper antifungal therapy, abstaining from using corticosteroids, and education are vital components in treating tinea incognito [11].

The emergence of antifungal resistance, particularly within the *T. mentagrophytes* complex, has become a major challenge in the treatment of tinea incognito [11-13]. Treatment failure and relapse have become increasingly common, leading to the use of higher doses, prolonged treatment courses, or combination antifungal therapy in resistant cases. Therefore, AFST and the judicious use of antifungal agents are strongly recommended. Early diagnosis, appropriate antifungal therapy, avoidance of corticosteroid misuse, and patient education remain essential components in the management of tinea incognito [11].

Limitations, recommendations, and future directions

It should be noted that this literature review uses only previously published data, which may have different designs, diagnostic criteria, and geographic distributions, thereby affecting their comparability. A considerable share of the information presented originates from studies conducted at a single center or specific region, particularly from tropical countries, which does not necessarily reflect the global disease prevalence. Besides, the lack of standardized diagnostic guidelines and different diagnostic procedures, including clinical diagnoses lacking mycological confirmation, could impact the reliability of results. Changing patterns of resistance and emerging epidemiology are likely underestimated due to insufficiently long and multicentric studies.

Strict control measures over the over-the-counter ​​​​​sale of steroid combination creams are critical to eliminate the misuse of these products. Emphasis should be placed on the need for appropriate laboratory diagnosis, including KOH examination and fungal cultures, before initiating any kind of therapy. Patient education campaigns aimed at increasing public awareness about the adverse effects of inappropriate steroid use should become widespread. The development of treatment guidelines and implementation of antifungal stewardship programs may help minimize failure and recurrence rates.

Future studies should consider developing fast, accurate, and affordable diagnostic methods, such as molecular approaches, to promote prompt, effective diagnosis of dermatophytic infections. Large-scale multicentric epidemiological investigations are important to gain insights into the epidemiology, transmission, and resistance profiles related to steroid-induced dermatophytosis. In addition, exploration of new antifungal drugs and combination therapies will be necessary to tackle the issue of drug resistance. Public health efforts related to educating individuals and regulating topical steroids will also be essential to manage the disease effectively.

## Conclusions

In conclusion, steroid-modified dermatophytosis represents an evolving and significant clinical challenge, particularly in regions like India, where misuse of topical corticosteroid combinations is widespread. The inappropriate use of steroids alters the natural course of dermatophyte infections by suppressing host immune responses, masking classical clinical features, and promoting chronic, recurrent, and atypical presentations. These changes not only complicate clinical recognition but also reduce the sensitivity and reliability of conventional diagnostic methods such as microscopy and culture.

Furthermore, emerging species such as *T. indotineae* and increasing antifungal resistance highlight the urgent need for improved diagnostic strategies, including molecular techniques and AFST. However, the limited accessibility and high cost of advanced diagnostic methods remain barriers to their routine practice. Addressing this issue requires a multifaceted approach, including strict regulation of over-the-counter steroid-containing formulations, increased awareness among clinicians and patients, and adherence to evidence-based antifungal therapy. Strengthening laboratory support and promoting antifungal stewardship are also essential to curb the growing burden of recalcitrant dermatophytosis and improve patient outcomes.
